# Targeting N-Glycan Cryptic Sugar Moieties for Broad-Spectrum Virus Neutralization: Progress in Identifying Conserved Molecular Targets in Viruses of Distinct Phylogenetic Origins

**DOI:** 10.3390/molecules20034610

**Published:** 2015-03-12

**Authors:** Denong Wang, Jin Tang, Jiulai Tang, Lai-Xi Wang

**Affiliations:** 1 Tumor Glycomics Laboratory, SRI International Biosciences Division, Menlo Park, CA 94025, USA; E-Mail: jiafengjushi@live.com; 2 Department of Pediatrics, The First Affiliated Hospital of Anhui Medical University, Hefei 230032, China; E-Mail: tangjiulai8888@21cn.com; 3 Institute of Human Virology, Department of Biochemistry & Molecular Biology, University of Maryland School of Medicine, Baltimore, MD 21201, USA; E-Mail: LWang@ihv.umaryland.edu; 4 Department of Chemistry and Biochemistry, University of Maryland at College Park, College Park, MD 20742, USA

**Keywords:** carbohydrate microarrays, oligomannoses, AGOR, ASOR, GNA, 2G12, HIV-1, HCMV, SARS-CoV

## Abstract

Identifying molecular targets for eliciting broadly virus-neutralizing antibodies is one of the key steps toward development of vaccines against emerging viral pathogens. Owing to genomic and somatic diversities among viral species, identifying protein targets for broad-spectrum virus neutralization is highly challenging even for the same virus, such as HIV-1. However, viruses rely on host glycosylation machineries to synthesize and express glycans and, thereby, may display common carbohydrate moieties. Thus, exploring glycan-binding profiles of broad-spectrum virus-neutralizing agents may provide key information to uncover the carbohydrate-based virus-neutralizing epitopes. In this study, we characterized two broadly HIV-neutralizing agents, human monoclonal antibody 2G12 and Galanthus nivalis lectin (GNA), for their viral targeting activities. Although these agents were known to be specific for oligomannosyl antigens, they differ strikingly in virus-binding activities. The former is HIV-1 specific; the latter is broadly reactive and is able to neutralize viruses of distinct phylogenetic origins, such as HIV-1, severe acute respiratory syndrome coronavirus (SARS-CoV), and human cytomegalovirus (HCMV). In carbohydrate microarray analyses, we explored the molecular basis underlying the striking differences in the spectrum of anti-virus activities of the two probes. Unlike 2G12, which is strictly specific for the high-density Man_9_GlcNAc_2_Asn (Man9)-clusters, GNA recognizes a number of N-glycan cryptic sugar moieties. These include not only the known oligomannosyl antigens but also previously unrecognized tri-antennary or multi-valent GlcNAc-terminating N-glycan epitopes (Tri/m-Gn). These findings highlight the potential of N-glycan cryptic sugar moieties as conserved targets for broad-spectrum virus neutralization and suggest the GNA-model of glycan-binding warrants focused investigation.

## 1. Introduction

Developing broad-range virus-neutralizing antibodies (bnAbs) requires identifying the immunological targets that are conserved among the targeted viruses and are suitable for antibody targeting. Discovery of oligomannosyl moieties as targets of bnAbs against HIV-1 has stimulated substantial interest in carbohydrate moieties as vaccine candidates [1,2,3,4,5,6,7,8,9,10,11]. The heavy glycosylation of the HIV envelope with oligomannoses appears to be a defense mechanism for the virus to evade host immune recognition of protein-based neutralizing epitopes. However, isolation of a number of glycan-dependent HIV bnAbs from HIV-infected individuals, such as 2G12, PG9/PG16, and other potent bnAbs [1,5,6,10,12,13], strongly suggests that the “glycan shield” of HIV virions may also display important targets for immunological intervention against viral infections.

Like HIV-1, virtually all human viruses decorate their virions with carbohydrate moieties. Thus, the potential of viral carbohydrates as immunological targets for other viral pathogens warrants exploration. One intriguing question is whether human viruses of distinct phylogenetic origins, such as HIV-1 and SARS-CoV, may display conserved glycan targets that are suitable for broad virus neutralization. Availability of bnAbs targeting such conserved glycans is clearly important for improving a biodefensive public health response to unexpected viral epidemics.

In response to the 2003 SARS epidemic, our team introduced carbohydrate microarrays to study SARS-CoV-elicited anti-glycan antibodies [14]. In essence, we characterized the carbohydrate-binding activity of the SARS-CoV-neutralizing antibodies elicited by immunizing horses with an inactivated SARS-CoV vaccine. In these horse antibodies, we detected unexpected auto-antibody reactivity specific for the carbohydrate moieties of an abundant human serum glycoprotein, asialo-orosomucoid (ASOR). The targeted carbohydrates are tri-antennary type II (Galβ1→4GlcNAc) or multi-valent type II (Tri/m-II) sugar moieties.

The N-glycosylation pathway has potential to generate numerous cryptic N-glycans of distinct structural characteristics. The spike glycoprotein of SARS-CoV has 23 potential N-linked glycosylation sites [15,16,17,18]. Ritchie *et al.* determined the glycan profiles of the spike protein produced by monkey Vero-E6 cells [19]. Its major glycans include high-mannose (Man_5-9_GlcNAc_2_), hybrid, and bi-, tri- and tetra-antennary complex carbohydrates with and without bisecting GlcNAc and core fucose. Interestingly, sialylation was negligible in the spike proteins produced by monkey Vero-E6 cells, which led to exposure of terminal galactoses in Tri/m-II glyco-determinants. Thus, induction of anti-ASOR auto-antibodies by inactivated SARS-CoV can be attributed to the fact that ASOR and the SARS-CoV spike glycoprotein commonly express the Tri/m-II cryptic glyco-determinants. Of note, this structural glycomics study revealed that SARS-CoV also expresses the high-mannose series of carbohydrate structures as its major glycan moieties. Thus, despite the fact that the two viruses differ in their general glycan profiles, they commonly overexpress oligomannosyl cores of N-glycans.

A significant number of virus-neutralizing agents target oligomannosyl moieties. Notably, these include mAb 2G12 and lectin GNA. However, the two probes represent distinct classes of virus-neutralizing agents. The former is “mono-specific” for HIV-1; the latter is “pauci reactive”, being a potent neutralizer for several viruses, including at least HIV-1, HCMV, and SARS-CoV [20,21,22,23,24]. Given the spectrum of anti-virus activities, GNA may serve as a model for exploration of broad-spectrum virus-neutralizing epitopes. An essential step toward this goal is to identify the natural ligands of GNA that are preserved among GNA-targeted viral pathogens, which is the focus of this study.

## 2. Results and Discussion

We reasoned that exploring glycan-binding profiles of broadly virus-neutralizing agents may provide key information to uncover the carbohydrate-based viral neutralization epitopes. In the first set of the experiments, we verified that the preparations of GNA or 2G12 we utilized recognize corresponding epitopes presented by the native viral antigens. Subsequently, we performed a comparative carbohydrate microarray analysis to characterize the glycan-binding profiles of 2G12 and GNA and to pinpoint specific glyco-epitopes they recognize.

### 2.1. Detection of GNA- or 2G12-Epitopes in the Native Viral Antigen Preparations

**Figure 1:**
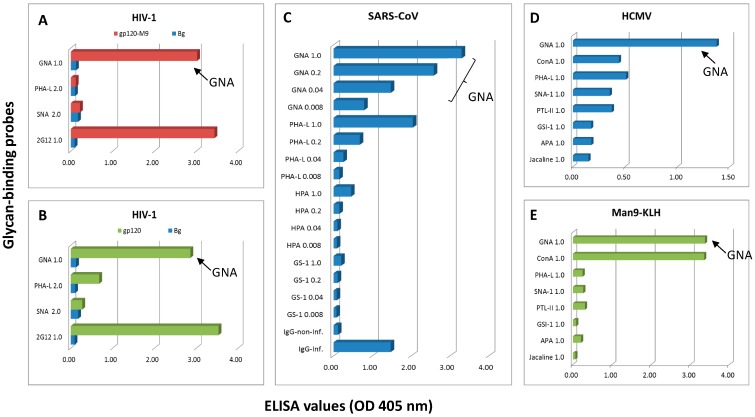
ELISA assays validated GNA-epitope expression by native viral antigens derived from HIV-1, SARS-CoV, or HCMV. (**A**,**B**) HIV-1-specific ELISA with Bal-gp120-Man9 (A) or Bal-gp120 (B) coated at 1.0 µg/mL; (**C**) SARS-CoV-specific ELISA, which was optimized for preserving native viral antigens; (**D**) HCMV-specific ELISA with purified virus at 1.0 µg/mL; and (**E**) Man9-KLH conjugate was applied at 10.0 µg/mL for coating. ELISA results were presented without background subtraction in **A**–**C** or with background subtraction in D and E. Lectins were applied at the concentrations (µg/mL) as specified. The ELISA data shown here are representative results of multiple assays.

**Figure 2:**
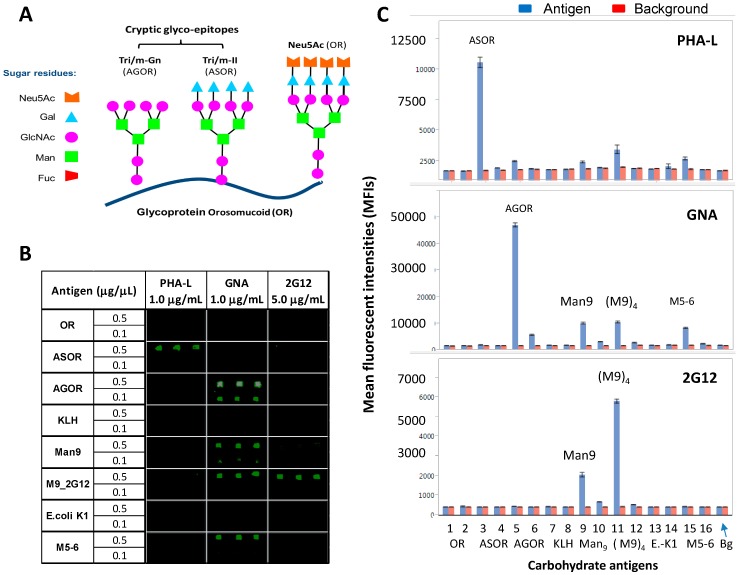
A comparative microarray analysis of the glyco-epitopes that are recognized by PHA-L, GNA, and 2G12. (**A**) Schematic of N-glycan “cryptic” glyco-epitopes, Tri/m-II and Tri/m-Gn, that are displayed by autoantigen, ASOR and AGOR, respectively; (**B**) Microarray images of PHA-L, GNA, or 2G12 staining against autoantigens, OR, ASOR, AGOR, Man9, (M9)_4_, and Ribonuclease B, which predominately express Man5 and Man6 (M5-6), as well as control probes, KLH, and *E. coli* K1. *Importantly, GNA binds to AGOR but not to ASOR or OR*; and (**C**) Microarray datasets for PHA-L (**Upper**), GNA (**Middle**), and 2G12 (**Bottom**), respectively. Each antigen was spotted in triplicate at given concentrations as specified. Results were compared using overlay plots of the MFIs of staining signal (*blue bars*) versus those of local backgrounds surrounding the antigen microarrays (*red bars*).

In Figure 1A,B, we examined detection of GNA- and 2G12-epitopes in HIV-1 gp120 glycoproteins. To preserve the native glyco-epitopes, we produced two gp120 preparations in HEK293 cells, *i.e.*, Bal-gp120-Man9 and Bal-gp120. Bal-gp120-Man9 was expressed in HEK293 cells in the presence of kifunensine (2 µg/mL), a potent α-mannosidase I inhibitor, following the previously described procedures [25,26]. The use of the α-mannosidase I inhibitor allows the enrichment of high-mannose type (Man9) glycoform through blocking further glycosylation processing to complex or hybrid carbohydrates. Thus, Bal-gp120-Man9 was produced to resemble the native envelope spikes of HIV, which are almost entirely coated with oligomannose antigens [27]. These proteins were applied on ELISA plates at 1.0 µg/mL, and the glycan staining was performed in comparison with lectins Phaseolus vulgaris-L lectin (PHA-L) and Sambucus nigra I agglutinin (SNA-I). With GNA and 2G12 targeting oligomannosyl epitopes, PHA-L for Tri/m-II cryptic epitopes, and SNA-I recognizing α2-6-linked Neu5Ac residues, this panel of probes monitors expression of layers of cryptic glyco-epitopes as schematically shown in Figure 2A.

This assay demonstrated that both Bal-gp120-Man9 (Figure 1A) and Bal-gp120 (Figure 1B) were strongly positive with GNA and 2G12. PHA-L was selectively reactive with Bal-gp120 but not with Bal-gp120-Man9. This PHA-L-differential staining pattern reflects the effective blockage of biosynthesis of Tri/m-II complex moieties during Bal-gp120-Man9 production as we initially planned. SNA-1 was negative to both glycoproteins under the same staining condition.

We further examined whether SARS-CoV expresses the native GNA-epitopes and PHA-L-epitopes. In Figure 1C, the ELISA plates were coated with the native SARS-CoV antigens that were expressed by Vero E6 cells. These plates were treated with a disinfecting fixing agent and gamma-irradiated to inactivate infectious virus particles while preserving antigenic structures of SARS-CoV. Lectins were applied at the concentrations specified in the figures.

This assay demonstrates that the PHA-L-epitopes were readily detected in the SARS-CoV-coated ELISA plates as we previously observed [14]. Moreover, it shows that GNA is strongly positive with SARS-CoV. By contrast, other Gal/GalNAc-reactive lectins, including *Helix pomatia* (HPA) and *Griffonia (Bandeiraea) simplicifolia*-I (GS-I), were only marginally reactive with SARS-CoV antigens. HPA is specific for GalNAc- and O-GlcNAc-moieties [28]; GS-I recognizes the α-Gal-epitope [29,30]. The latter is conserved in many microorganisms but is negligible in SARS-CoV as expected. 

In Figure 1D, we examined whether HCMV expresses oligomannosyl epitopes using GNA and concanavalin A (Con A) in comparison with other lectins of different specificities. In this experiment, ELISA was coated with purified virus (AD169, Creative Diagnostics, New York, NY, USA) at protein concentration approximately 1.0 µg/mL for lectin-screening. The two anti-mannose agents differ in epitope-binding specificities. GNA is specific for the Manα1,3Man and Manα1,6Man moieties of oligomannoses; Con A recognizes terminal Manα1**→**moieties that are more broadly expressed by mannose-containing antigens. Figure 1E illustrates lectin-binding of a synthetic glycoconjugate, Man9-KLH, which bears both GNA- and Con-A-glyco-epitopes [31]. Inspection of Figure 1D readily reveals that the relative binding activity of GNA is markedly higher than the Con-A activity in the HCMV-ELISA assay although the two mannose-specific lectins are similarly reactive with the Man9-KLH standard in the assay. Other lectins of different glycan-binding specificities show certain reactivities with HCMV lysates, which may reflect the glycome complexity of this most intricate human virus [32,33], which requires further study.

In summary, we verified expression of the native GNA-epitopes by three phylogenetically distinct viruses, HIV-1, SARS-CoV, and HCMV. In the context of a panel of lectins of different specificities, GNA was identified to have the highest relative binding activities with SARS-CoV and HCMV.

### 2.2. Carbohydrate Microarrays to Explore the Potential Glyco-Epitopes of GNA

The ability of GNA to bind and neutralize viruses of distinct phylogenetic origins unavoidably raises questions about its glycan-binding specificity and potential cross-reactivity. One plausible explanation is that these targeted viruses commonly express the defined GNA-epitopes of Manα1,3Man and Manα1,6Man moieties. However, the selective GNA-positive staining of HCMV without parallel Con A reactivity (Figure 1D) suggests an alternative possibility, *i.e.*, presence of other carbohydrate moieties that are negative or weakly reactive with Con A but strongly reactive with GNA. A carbohydrate antigen with such differential reactivity between GNA and Con A is a yeast-derived phosphomannan polysaccharide (P-Man), which is nevertheless not present in the viral glycome [31,34].

**Figure 3:**
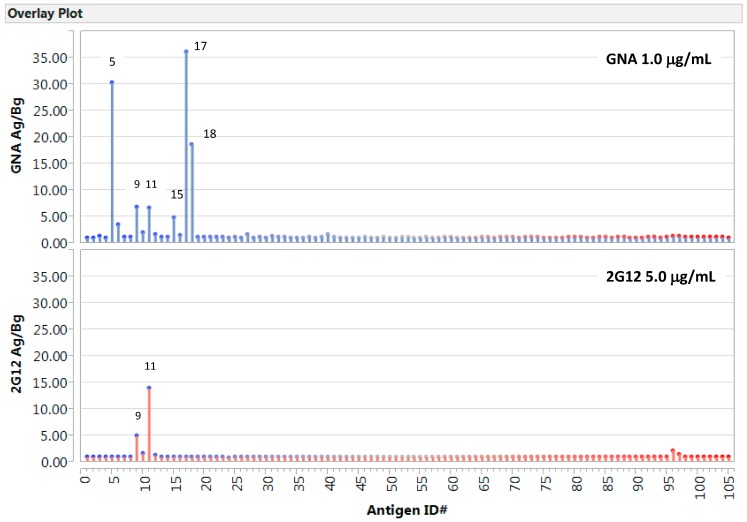
Carbohydrate microarrays reveal distinct models of glycan recognition by GNA and 2G12, respectively. Antigens spotted include carbohydrates (1–39), lipids/liposomes (40–91), and proteins (92–104). (**Upper panel**) GNA stain (1.0 µg/mL); (**Bottom panel**) 2G12 stain (5.0 µg/mL). Results were plotted as antigen-specific reading over background. Corresponding microarray datasets are shown in Supplementary Table S2.

To support exploration of the potential GNA glyco-epitopes in this study, we produced a set of comprehensive antigen microarrays, which include a large-panel of carbohydrates, lipids/liposomes, and protein antigens (Supplementary Table S1). Key compounds supporting glyco-epitope analysis include the following:

(1) Orosomucoid (OR) (Neu5Ac), ASOR (Tri/m-II), and Agalacto-OR (AGOR) (Tri/m-Gn) (Figure 2A). These autoantigens display distinct glyco-epitopes with identical protein carriers. ASOR is an asialo-derivative of OR, and AGOR is an agalacto-derivative of ASOR. They are crucial for defining binding-specificities for N-glycan cryptic epitopes, Tri/m-II, and Tri/m-Gn.
(2) Thiolated keyhole limpet hemocyanin (KLH-SH), (Man9GlcNAc2Asn)n-KLH (Man9), and [(Man9GlcNAc2Asn)4]n-KLH (M9_2G12). The two Man9-KLH conjugates display Man9 moieties in two defined cluster configurations. M9_2G12 is highly specific for 2G12.
(3) Ribonuclease B (RB) with Man5-6GlcNAc2Asn (M5-6) as the main glycans.
(4) Phosphomannan (P-Man), a yeast polysaccharide. Both M5-6-RB and P-Man are known to be positive with GNA; the former but not the latter also binds to Con A. Using these microarrays, we characterized GNA, PHA-L, and 2G12 for their epitope-binding profiles. Results are summarized in Figure 2 and Figure 3.

Figure 2A is a schematic view of structural relationship among autoantigens, OR, ASOR, and AGOR and the glyco-epitopes they display. Figure 2B shows microarray images of PHA-L, GNA, or 2G12 staining against a panel of autoantigens, including OR, ASOR, AGOR, Man9, (M9)_4_, which stands for [(Man9GlcNAc2Asn)4]n-KLH (M9_2G12) conjugates, and M5-6-RB. The two control probes spotted are KLH and *E. coli* K1 polysaccharide. Microarray datasets corresponding to Figure 2B are shown in Figure 2C. As illustrated, each antigen was spotted in triplicate at given concentrations as specified. Microarray detections are shown as the mean fluorescent intensities (MFIs) of triplicate microspots for the arrays stained with PHA-L, GNA, and 2G12, respectively. Results were compared using overlay plots of the MFIs of staining signal (*blue bars*) *versus* those of local backgrounds surrounding the antigen microarrays (*red bars*).

Figure 2B,E show that PHA-L and 2G12 are specific for ASOR (Tri/m-II) and (M9)_4_, respectively, which is expected. However, GNA highly and selectively binds to a number of N-glycan cryptic sugar moieties, including Man9, (M9)_4_, M5-6, and AGOR. GNA-binding of AGOR is a novel observation. Given that GNA had no binding to ASOR (Tri/m-II), which only differs from AGOR (Tri/m-Gn) by having the terminal galactoses. This result demonstrates, therefore, that GNA is most likely specific for the Tri/m-Gn-glyco-epitopes of AGOR with exposed terminal GlcNAc moieties.

In Figure 3 below, we illustrate the global antigen-binding profiles of GNA and 2G12 against the full panel of antigen preparations. Microarray results were plotted as the ratio of antigen-specific signals over the corresponding spots’ background readings, *i.e.*, GNA Ag/Bg, or 2G12 Ag/Bg. To assist a comparative analysis of binding profiles between GNA and 2G12, datasets were plotted in the same scale in the y-axis. The positive detections labeled with numbers in the graphs are AGOR (5), Man9 (9), (M9)_4_ (11), M5-6-RB (15), and P-Man in two dilutions (17 and 18). The whole datasets for these graphs are listed in Supplementary Table S2.

In this microarray analysis, GNA and 2G12 show minimal or no cross-relativities with irrelevant antigens but illustrate strikingly different glycan-binding profiles. 2G12 is “mono-specific” for Man9-clusters (9 and 11) as expected. By contrast, GNA is “pauci reactive” with a number of glycan targets. Its binding to the spotted oligomannose antigens (5, 9, 11, and 15) and P-Man (17 and 18) can be attributed to the known GNA-specificity for recognition of the shared Manα1,3Man and/or Manα1,6Man moieties. However, GNA-binding to AGOR, but not to ASOR or OR, indicates that GNA also specifically recognize the Tri/m-Gn-glyco-determinants displayed by AGOR. Supporting to this prediction is the fact that AGOR, ASOR, and OR are negative to other anti-mannose agents, including lectin Con A, mAbs 2G12, TM10, and polyclonal antisera that recognize oligomannosyl moieties of varies cluster configurations [31,35]. Molecular mechanisms underlying GNA-recognition of the Tri/m-Gn-determinants of AGOR are yet to be explored.

## 3. Experimental Section

### 3.1. Printing Protein, Carbohydrate and Lipid/Liposome Microarrays

A high-precision microarray robot (PIXSYS 5500C, Cartesian Technologies, Irvine, CA, USA) was used to spot antigen preparations onto glass slides pre-coated with nitrocellulose polymer (FAST Slides; Schleicher & Schuell, Keene, NH, USA) as described [36]. The antigen preparations applied include carbohydrates, proteins/peptides, and liposomes of various compositions as listed in Supplementary Table S1. Proteins and carbohydrates were dissolved in phosphate-buffered saline (PBS; pH 7.4) and saline (0.9% NaCl), respectively. Liposome preparations were suspended in saline (0.9% NaCl) at the concentrations specified and were printed in triplicate with spot sizes of ~150 µm and at 375-µm intervals, center to center. The printed microarrays were air-dried and stored at room temperature without desiccant before application. 

### 3.2. Staining and Scanning of Microarrays

Immediately before use, the printed microarrays were rinsed with PBS, pH 7.4, with 0.05% (v/v) Tween 20 and then blocked by incubating the slides in 1% (w/v) bovine serum albumin (BSA) in PBS containing 0.05% (w/v) NaN_3_ at room temperature (RT) for 30 min. They were then incubated at RT with 2G12 (NIH AIDS Reagent Program, Germantown, MD, USA), biotinylated PHA-L (PHA-L^BI^), or GNA^BI^ (EY Laboratories, Inc., San Mateo, CA, USA) at an indicated titration in 1% (w/v) BSA in PBS containing 0.05% (w/v) NaN_3_ and 0.05% (v/v) Tween 20. The secondary antibodies or streptavidin conjugates applied for microarray staining are specified in the figure legends. The stained slides were rinsed five times with PBS with 0.05% (v/v) Tween 20, air-dried at room temperature, and then scanned for fluorescent signals using a ScanArray5000A Microarray Scanner (PerkinElmer Life Science, Boston, MA, USA) following the manufacturer’s manual.

### 3.3. Microarray Data-Processing and Statistical Analysis

Fluorescence intensity values for each array spot and its background were calculated using ScanArray Express software (PerkinElmer Life Science, Boston, MA, USA). SAS Institute’s JMP-Genomics software package (Cary, NC, USA) was used for further microarray data processing and statistical analysis. In Figure 2, microarray detections are shown as the mean fluorescent intensities (MFIs) of triplicate detections captured by ScanArray 5000A for the arrays stained with PHA-L, GNA, or 2G12. Analysis of such microarray data in association with visual inspection of the microarray image provided an evaluation of reproducibility and variation of this antigen microarray technology. In Figure 3, microarray results were plotted as the ratio of antigen-specific signal over corresponding spots’ background reading, *i.e.*, GNA Ag/Bg, or 2G12 Ag/Bg. The whole microarray datasets for PHA-L, GNA, and 2G12 were presented in Supplementary Table S2.

### 3.4. Viral Antigen Preparations and Antigen-Specific ELISA

Two HIV-1 gp120 preparations, Bal-gp120-Man9 and Bal-gp120, were produced in HEK293 cells as previously described [25,26]. Bal-gp120-Man9 was expressed in the presence of the α-mannosidase I inhibitor, kifunensine (2 µg/mL), to enrich high-mannose type glycoforms. A preparation of sucrose density-gradient-purified HCMV (AD169) was obtained from Creative Diagnostics (NY, NY, USA). An ELISA protocol described previously [37] for detection of anti-carbohydrate antibodies was followed with minor modifications. In Figure 1A–E, antigen preparations were diluted in 0.1 M sodium bicarbonate buffer solution, pH 9.6, for coating on ELISA microplates (NUNC, MaxiSorp, Thermo Fisher Scientific Inc., Santa Clara, CA, USA) followed by blocking using 1% BSA, PBST. In Figure 1C, a SARS-CoV-specific ELISA kit (EUROIMMUN AG, Lübeck, Germany) was used to measure SARS-CoV-expression of lectin-specific glyco-epitopes. Anti-glycan antibodies or biotinylated lectins were pre-titrated in 1% BSA, PBST for ELISA. The bound antibodies were revealed by an alkaline phosphatase (AP)-conjugate of goat anti-human IgG-Fc-specific antibody, and the biotinylated lectins captured were quantified by AP-streptavidin-conjugate.

## 4. Conclusions

In this pilot study, we examined whether human viruses of distinct phylogenetic origins may express common carbohydrate moieties. Using carbohydrate microarrays and ELISA-based viral glycan-profiling analysis, we characterized two broadly HIV-neutralizing agents, human monoclonal antibody 2G12 and lectin GNA. Although these agents were known to target oligomannosyl antigens, they differ strikingly in the spectrum of viruses they effectively neutralize. The former is solely HIV-specific; the latter is broadly reactive with human viruses, including HIV-1, SARS-CoV, and HCMV, that are phylogenetically and pathogenically distinct. Our carbohydrate microarray analyses demonstrate the two probes differ strikingly in glycan-targeting specificities and the spectrum of glyco-epitopes they recognize. Although 2G12 is strictly specific for the high-density Man9 clusters that decorate the HIV envelope spike, GNA recognizes a number of N-glycan cryptic sugar moieties. These include oligomannoses and the previously unrecognized Tri/m-Gn-glyco-determinants. The latter appear to be the potent natural ligands of GNA. Molecular mechanisms underlying the GNA-model of “pauci reactive” glycan-binding and broad virus-neutralization warrant further investigation. Owing to the potential immunogenic activity as a plant-derived lectin, GNA is unlikely suitable for anti-virus therapy *in vivo*. Thus, effort must also be made to establish GNA-like potent and broadly virus-neutralizing antibodies, especially humanized or fully human mAbs that are readily applicable in the front-line biodefense against emerging viral pathogens.

## Supplementary Materials

Supplementary materials can be accessed at: http://www.mdpi.com/1420-3049/20/03/4610/s1.

## Abbreviations

OR: (orosomucoid)
ASOR: (asialo-orosomucoid)
AGOR: (agalacto-orosomucoid)
Tri/m-II: (Tri-antennary and multivalent type II (Galβ1→4GlcNAc) chain epitopes)
Tri/m-Gn: (Tri-antennary or multi-valent GlcNAc-terminating epitopes)
GNA: (Galanthus nivalis agglutinin)
PHA-L: (Phaseolus vulgaris-L lectin)
SNA-I: (Sambucus nigra I agglutinin)
HCMV: (human cytomegalovirus)
HIV-1: (human immunodeficiency virus-1)
SARS-CoV: (severe acute respiratory syndrome coronavirus)
