# Supplementary material for: Targeting N-Glycan Cryptic Sugar Moieties for Broad-Spectrum Virus Neutralization: Progress in Identifying Conserved Molecular Targets in Viruses of Distinct Phylogenetic Origins
Source: Molecules. 2015 Mar 12;20(3):4610–22. doi: 10.3390/molecules20034610 (PMC4633014; doi:10.3390/molecules20034610)
Supplement: Supplementary file 1 [file molecules-20-04610-s001.zip › Supplementary Table S1.pdf]

Supplementary Materials

Table S1. Antigen preparations used in microarray studies.

Antigen Name	Antgen Preparations *	Description	Source	References
OR	OR, 0.5 mg/mL	Orosomucoid (human a1-acid glycoprotein)	Sigma-Aldrich	[1]
ASOR	ASOR, 0.5 mg/mL	Asialo-orosomucoid	From the late Prof. Elvin A. Kabat (Columbia University)	[1]
AGOR	AGOR, 0.5 mg/mL	Agalacto-orosomucoid	From the late Prof. Elvin A. Kabat (Columbia University)	[1]
KLH-SH	KLH-SH, 0.5 mg/mL	Thiolated keyhole limpet hemocyanin	Present authors	[2]
Man9	Man9-KLH, 0.5 mg/mL	$(Man9GlcNAc2Asn)_n$-KLH	Present authors	[2]
M9(2G12)	Man9-Cluster(2G12)-KLH, 0.5 mg/mL	$[(Man9GlcNAc2Asn)_4]_n$-KLH	Present authors	[2]
E. coli. K1	E. coli. K1 CP, 0.5 mg/mL	E. coli K1 capsular polysaccharide (CP)	From the late Prof. Elvin A. Kabat (Columbia University)	[3]
Man5_6-RB	Man 5-6 RB, 0.5 mg/mL	Ribonuclease B with $Man_{5-6}GlcNAc_2Asn$ as the main glycans (pancreas, bovine)	Sigma-Aldrich	[4]
P-Man	B2448, 0.5 mg/mL	Phosphomannan, NRRL B-2448	Northern Regional research laboratory, (Peoria, IL) NRRL B-2448	[3]
B1299S	B1299S, 0.5 mg/mL	α(1→6)dextran, NRRL B-1299S	Northern Regional research laboratory, (Peoria, IL) NRRL B-2448	[5]
B1355S	B1355S, 0.5 mg/mL	α(1→3)(1→6)dextran, NRRL B-1355S	Northern Regional research laboratory, (Peoria, IL) NRRL B-2448	[5]
Levan	Levan, 0.5 mg/mL	Levan (from preparation of B-512-E dextran)	Commercial Solvents Co. (Terre Haute, IN)	[3]
N279	N279, 0.5 mg/mL	α(1→6)dextran, NRRL N279	Northern Regional research laboratory, (Peoria, IL) NRRL B-2448	[5]

Table S1. *Cont.*

Antigen Name	Antgen Preparations *	Description	Source	References
Bacto-Agar	Bacto-Agar, 0.5 mg/mL	Bacto-agar 20°C extract	From the late Prof. Elvin A. Kabat (Columbia University)	[6]
E. coli. 5014	E. coli. LPS 5014, 0.5 mg/mL	Lipopolysaccharides (rough strains) from Escherichia coli J5 (Rc mutant)	Sigma-Aldrich	[4]
E. coli. 2630	E. coli. LPS 2630, 0.5 mg/mL	Lipopolysaccharides from Escherichia coli 0111:B4	Sigma-Aldrich	[4]
E. coli. K100	E. coli. K100 CP, 0.5 mg/mL	E. coli K100 capsular polysaccharide (CP)	From the late Prof. Elvin A. Kabat (Columbia University)	[3]
E. coli. K92	E. coli. K92 CP, 0.5 mg/mL	E. coli K92 capsular polysaccharide	From the late Prof. Elvin A. Kabat (Columbia University)	[3]
S. dysenterine type 1	S. dysenterine type I O-SP, 0.5 mg/mL	Shigella dysenteriae Type 1 O-specific polysaccharide	From Drs. John Robbins and Rachel Schneerson (NICHD, NIH)	[7]
S.typhi LPS	S.typhi LPS7261, 0.5 mg/mL	Lipopolysaccharides from Salmonella enterica serotype typhimurium L7261	Sigma-Aldrich	[4]
Sulfatide	Sulfatide, 2.0 mg/mL	Sufatides (Cerebroside sulfates), (Brain, Porcine)	Avanti Polar lipids, Alabaster, AL, USA	[4]
Ganglioside	Ganglioside, 2.0mg/mL	Total Ganglioside-Ammonium Salt, (Brain, Porcine)	Avanti Polar lipids, Alabaster, AL, USA	[4]
Ceremide	Ceremide, 2.0 mg/mL	Ceramide (Brain, Porcine)	Avanti Polar lipids, Alabaster, AL, USA	[4]
DIMPS	DIMPS, 2 mg/mL	1,2-Dimyristoyl-sn-Glycero—3—[phospho-L—serine](Sodium Salt) (DMPS)	Avanti Polar lipids, Alabaster, AL, USA	[4]
GM1/PTC_0.02/2.0	GM1/PTC, 0.02 mg/2 mg/mL, 4 °C, 24 h	GM1 Ganglioside-Sodium Salt (Brain, Ovine)	Avanti Polar lipids, Alabaster, AL, USA	[4]
GM1/PTC_0.2/2.0	GM1/PTC, 2 mg/2 mg/mL, 4 °C, 14 Months	GM1 Ganglioside-Sodium Salt (Brain, Ovine)	Avanti Polar lipids, Alabaster, AL, USA	[4]

Table S1. *Cont.*

Antigen Name	Antgen Preparations *	Description	Source	References
Ganglioside/PTC_0.2/2.0	Ganglioside/PTC, 0.2 mg/2 mg/mL, 4 °C, 24 h	Total Ganglioside Extract (Brain, Porcine-Ammnium Salt)	Avanti Polar lipids, Alabaster, AL, USA	[4]
Erythrosphingosine/PTC_0.2/2.0	Erythrosphingosine/PTC, 0.2mg/2.0mg/mL, 4°C, 24 h	D-erythro- Sphingosine (Egg, Chicken)	Avanti Polar lipids, Alabaster, AL, USA	[4]
Phytosphingosine/PTC_0.2/2.0	Phytosphingosine/PTC, 0.2 mg/2.0 mg/mL, 4 °C, 24 h	Phytosphingosine 4-Hydroxysphinganine (Saccharomyces cervisiae)	Avanti Polar lipids, Alabaster, AL, USA	[4]
Glucocerebroside/PTC_0.2/2.0	Glucocerebroside/PTC, 0.2 mg/2 mg/mL, 4 °C, 24 h	Glucocerebrosides (Soy, 98%)	Avanti Polar lipids, Alabaster, AL, USA	[4]
Sulfatide/PTC_0.2/2.0	Sulfatide/PTC, 0.2 mg/2.0 mg/mL, 4 °C, 24 h	Sufatides (Cerebroside sulfates) (Brain, Porcine)	Avanti Polar lipids, Alabaster, AL, USA	[4]
Ceremide/PTC_0.2/2.0	Ceremide/PTC, 0.2 mg/2.0mg/mL, 4°C, 24 h	Ceramide (Brain, Porcine)	Avanti Polar lipids, Alabaster, AL, USA	[4]
Cerebroside/PTC_0.2/2.0	Cerebroside/PTC, 0.2 mg/2.0 mg/mL, 4 °C, 24 h	Total Cerebrosides (Brain, Porcine)	Avanti Polar lipids, Alabaster, AL, USA	[4]
DMPS/PTC_0.2/2.0	DMPS/PTC, 0.2mg/2.0mg/mL, 4 °C, 24 h	1,2-Dimyristoyl-sn-Glycero—3—[phospho-L—serine](Sodium Salt) (DMPS)	Avanti Polar lipids, Alabaster, AL, USA	[4]
Cardiolipin/PTC_0.4/2.0_D1	Cardiolipin/PTC, 0.4 mg/2 mg/mL, 4 °C, 24 h	Cardiolipin-Disodium Salt (heart, bovin)	Sigma Chemical Co., St. Louis, MO, USA	[4]
Cardiolipin/PTC_0.1/2.0 D1	Cardiolipin/PTC, 0.1 mg/2 mg/mL, 4 °C, 24 h	Cardiolipin-Disodium Salt (heart, bovin)	Sigma Chemical Co., St. Louis, MO, USA	[4]
Cardiolipin/PTC_0.4/2.0 4M	Cardiolipin/PTC, 0.4 mg/2 mg/mL, 4 °C, 4 Months	Cardiolipin-Disodium Salt (heart, bovin)	Sigma Chemical Co., St. Louis, MO, USA	[4]
Cardiolipin/PTC_0.1/2.0 4M	Cardiolipin/PTC, 0.1 mg/2 mg/mL, 4°C, 4 Months	Cardiolipin-Disodium Salt (heart, bovin)	Sigma Chemical Co., St. Louis, MO, USA	[4]
Cardiolipin/PTC_0.02/2.0 4M	Cardiolipin/PTC, 0.02 mg/2 mg/mL, 4 °C, 4 Months	Cardiolipin (C1649)	Sigma Chemical Co., St. Louis, MO, USA	[4]
aGal-T2/PTC_0.04/2.0	Galal,3Galb1-**HDPE/PTC, 0.04 mg/2 mg/mL, 4 °C, 12 Months	Galal,3Galb1-HDPE (aGal-terminal di-saccharides)	V-LABS, INC, Covington, LA, USA	[4]

Table S1. *Cont.*

Antigen Name	Antgen Preparations *	Description	Source	References
Lactosamine/PTC_0.04/2.0	Galbl,4GlcNACb1-HDPE/PTC, 4 °C, 12 Months	Galbl,4GlcNACb1-HDPE (Type II chain unit)	V-LABS, INC, Covington, LA, USA	[4]
aGal-T3/PTC_0.04/2.0	Galal,3Galbl,4GlcNACb1-HDPE/ PTC, 4 °C, 12 Months	Galal,3Galbl,4GlcNACbl-(aGal-terminal di-saccharides)-HDPE	V-LABS, INC, Covington, LA, USA	[4]
PLP139-150	PLP139-150, 1mg/mL	A synthetic peptide of human Myelin Proteolipid Protein (PLP), Amino acid residue 139-150	Present authors	[4]
PTC_D1 2.0	PTC 2.0 mg/mL, 4 °C, 24 h	L-a-Phosphatidylcholine (Egg, Chicken)	Avanti Polar lipids, Alabaster, AL, USA	[4]
PTC_4M 2.0	PTC 2.0 mg/mL, 4 °C, 4 Months	L-a-Phosphatidylcholine (Egg, Chicken)	Avanti Polar lipids, Alabaster, AL, USA	[4]
PTC_12M 2.0	PTC 2.0 mg/mL, 4 °C, 12 Months	L-a-Phosphatidylcholine (Egg, Chicken)	Avanti Polar lipids, Alabaster, AL, USA	[4]
PTC_14M 2.0	PTC 2.0 mg/mL, 4 °C, 14 Months	L-a-Phosphatidylcholine (Egg, Chicken)	Avanti Polar lipids, Alabaster, AL, USA	[4]
MBP1-11	MBP1-11, 1 mg/mL	A synthetic peptide of human myelin basic protein(MBP), N-terminal amino acid 1-11	Present authors	[8]
mMBP	mMBP, 0.5 mg/mL	Myelin Basic Protein (Brain, Murine)	ACCURATE CHEMICAL & SCIENTIFIC CORPORATION, WESTBURY, NY, USA	[8]
hMBP	hMBP, 0.5 mg/mL in 10 mM HCl	Myelin Basic Protein, iodination grade (Brain, Human)	ACCURATE CHEMICAL & SCIENTIFIC CORPORATION, WESTBURY, NY, USA	[8]
M0G	M0G, 1 mg/mL	Myelin Oligodendrocyte Glycoprotein (Brain, Human)	ACCURATE CHEMICAL & SCIENTIFIC CORPORATION, WESTBURY, NY, USA	[8]

* The antigen concentrations for initial microarray spotting were listed. The subsequent dilutions of each antigens were specified in Supplementary Table S2, Figures 2 and 3;

** HDPE: Neoglycolipids-1,2-di-O-hexadecyl-sn-glycero-3—phosphoethanolamine.

References

1. Wang, D.; Lu, J. Glycan arrays lead to the discovery of autoimmunogenic activity of SARS-CoV. *Physiol. Genomics* **2004**, *18*, 245–248. *Physiol. Genomics* **2004**, *18*, 245–248.
2. N, J.; Song, H.; Wang, Y.; Stamatos, N.M.; Wang, L.X. Toward a carbohydrate-based HIV-1 vaccine: Synthesis and immunological studies of oligomannose-containing glycoconjugates. *Bioconjug. Chem*. **2006**, *17*, 493–500.
3. Kabat, E.A.; Nickerson, K.G.; Liao, J.; Grossbard, L.; Osserman, E.F.; Glickman, E.; Chess, L.; Robbins, J.B.; Schneerson, R.; Yang, Y.H. A human monoclonal macroglobulin with specificity for alpha(2----8)-linked poly-N-acetyl neuraminic acid, the capsular polysaccharide of group B meningococci and Escherichia coli K1, which crossreacts with polynucleotides and with denatured DNA. *J. Exp. Med.* **1986**, *164*, 642–654.
4. Wang, D.; Bhat, R.; Sobel, R.A.; Huang, W.; Wang, L.X.; Olsson, T.; Steinman, L. Uncovering Cryptic Glycan Markers in Multiple Sclerosis (MS) and Experimental Autoimmune Encephalomyelitis (EAE). *Drug Dev. Res.* **2004**, *75*, 172–188.
5. Wang, D.; Liu, S.; Trummer, B.J.; Deng, C.; Wang, A. Carbohydrate microarrays for the recognition of cross-reactive molecular markers of microbes and host cells. *Nat. Biotechnol.* **2002**, *20*, 275–281.
6. Duckworth, M.; Yaphe, W. The structure of agar: Part I. Fractionation of a complex mixture of polysaccharides. *Carbohydr. Res.* **1971**, *16*, 189–197.
7. Pozsgay, V.; Kubler-Kielb, J.; Schneerson, R.; Robbins, J.B. Effect of the nonreducing end of Shigella dysenteriae type 1 O-specific oligosaccharides on their immunogenicity as conjugates in mice. *Proc. Natl. Acad. Sci. USA* **2007**, *104*, 14478–14482.
8. Robinson, W.H.; DiGennaro, C.; Hueber, W.; Haab, B.B.; Kamachi, M.; Dean, E.J.; Fournel, S.; Fong, D.; Genovese, M.C.; de Vegvar, H.E.; *et al*. Autoantigen microarrays for multiplex characterization of autoantibody responses. *Nat. Med*. **2002**, *8*, 295–301.
